# Erythropoiesis-stimulating agents: benefits and risks in supportive care of cancer

**DOI:** 10.3747/co.2008.172

**Published:** 2008-01

**Authors:** B.L. Melosky

**Keywords:** Erythropoiesis-stimulating agents, transfusion, quality of life, thrombosis, survival, tumour progression

## Abstract

Anemia, already common in cancer patients, is often exacerbated by chemotherapy. Cancer patients who are anemic have been shown to have a blunted response for production of endogenous erythropoietin growth factor. This anemia can be corrected with exogenous erythropoietin growth factors, of which three available are worldwide: epoetin alfa, epoetin beta, and darbepoetin alfa. Collectively, these drugs are known as erythropoiesis-stimulating agents (esas). Orders for esas have been used not only to reverse anemia so as to avoid blood transfusion, but also to improve quality of life. Guidelines have been developed for initiation, dosage titration, and termination of these agents. Since the late 1990s, trials have been conducted using esas in unapproved dosing regimens or to reach hemoglobin levels outside of approved guidelines, raising several safety concerns. The present article explores the risks and benefits of esas.

## 1. INTRODUCTION

Anemia is a common finding in cancer patients and can be a result of treatment or of underlying disease 1. A large-scale audit of blood transfusions in 2719 cancer patients showed that 38% had a hemoglobin level below 11.0 g/dL during chemotherapy and that 33% of the patients required at least one blood transfusion.^2^.

Recombinant human erythropoietin was first used clinically in renal dialysis patients in the 1980s, at which time it was shown to correct anemia in end-stage renal disease^3^. Those findings led to a recognition that anemia in patients with malignancy might also be able to be reversed, leading to an improvement in hemoglobin level, rate of transfusion, and quality of life (qol).

The first randomized trial in cancer patients demonstrated that recombinant human erythropoietin is effective for treating anemia in cancer patients on chemotherapy^4^. Three large, open, nonrandomized trials confirmed that finding and, more importantly, established that the highest incremental improvement in qol was achieved at an optimum hemoglobin level of 12 g/dL 5–7. That level of 12 g/dL is now considered the accepted level by various guidelines committees, including those of the American Society of Hematology 8, the American Society of Clinical Oncology 9, and the National Comprehensive Cancer Network 10. (The 12 g/dL level will be important to remember in the exploration of publications reporting negative safety and survival in the remainder of the present article.)

After the open-label studies, a randomized placebo-controlled study by Littlewood *et al.* 11 confirmed the relationship between anemia, fatigue, and qol. A retrospective review in that trial suggested a survival advantage in patients treated with epoetin alfa. That finding led to prospective trials that attempted to validate the better-survival hypothesis. To strengthen the argument, higher-than-normal hemoglobin levels were defined for achievement. As well, to broaden the indications for use, trials were conducted in patients not on active chemotherapy treatment. Neither of those strategies worked, and confusion regarding the safety of this class of drugs instead resulted. To complicate the data even more, the media have been emphasizing the profit margins of the pharmaceutical companies managing the drugs.

The aim of the present article is to critically review the benefits and risks associated with the use of erythropoiesis-stimulating agents (esas) as supportive care in cancer patients. Demonstrated benefits of esas include lower rates of transfusion and improved qol, including reduced fatigue and better cognition. Demonstrated risks of esas include risk of thromboembolic disease, negative impact on survival, and risk of tumour proliferation.

## 2. BENEFITS

### 2.1 Lower Rates of Transfusion

Two esas, epoetin alfa (Eprex: Janssen–Ortho, Toronto, ON) and darbepoetin alfa (Aranesp: Amgen, Thousand Oaks, CA, U.S.A.) are approved in Canada. The basis for this approval by the Health Protection Branch of Health Canada and the U.S. Food and Drug Administration (fda) was a proven reduction (as compared with placebo) in the proportion of patients transfused during chemotherapy.

Six randomized placebo-controlled double-blind clinical trials using epoetin alfa were pooled 12. Anemic cancer patients (*n* = 131) who were to receive at least 12 weeks of concurrent chemotherapy were randomized (1:1) to receive epoetin alfa 150 U/kg or placebo subcutaneously three times weekly for 12 weeks. Half of the patients received cisplatin-containing chemotherapy regimens. Efficacy results showed a reduction in transfusions by approximately 50% during the second and third months of chemotherapy in the patients treated with epoetin alfa. Table I summarizes the results.

Approval of darbepoetin alfa for the treatment of anemia in cancer patients on chemotherapy was also based on a proven reduction in transfusions. A phase III double-blind placebo-controlled randomized (1:1) study of darbepoetin alfa was preformed in anemic patients with untreated non-small-cell or small-cell lung cancer who were to receive at least 12 weeks of platinum-containing chemotherapy 13. A total of 314 patients were randomized to darbepoetin alfa 2.25 μg/kg or placebo subcutaneously weekly. Patients were stratified by tumour type. As in the epoetin alfa trial, efficacy results for treatment with darbepoetin alfa showed an approximately 50% reduction in transfusions, a result that was significant from week 5 through the end of treatment. Table II summarizes the results.

The risks of blood transfusion must be understood. The most common serious risk of red cell transfusion is transfusion-related acute lung injury. The risk of this complication is estimated to be 1 in 432 whole-blood units to 1 in 557,000 red-blood-cell units 14. The risk of infection with human blood products changes with time and has improved since the late 1990s because of the application of careful screening techniques for antibodies against hiv, hepatitis b and c, and West Nile virus. In 1991, the risk of hiv was 1 in 493,000; in 2003, it was estimated at less than 1 in 1,000,000 15. But it is the unknown bacterium or virus that cannot be uncovered through testing. For that reason, the Kreever Commission recommended that “the operator of the blood supply system promote appropriate use of, and alternatives to, blood components and blood products. Blood components and blood products will never be without risk. The best way to reduce that risk is to reduce their use” 16. For liability reasons, hospital authorities ask doctors and their patients to sign consent forms acknowledging that the patient understands the risk of such procedures. It would be short-sighted and naïve to believe that another infectious agent will never be seen in human blood products.

In summary, esas are efficacious in reducing the need for blood transfusions in cancer patients on chemotherapy. Across several studies, approximately 50% of patients on chemotherapy without esas required transfusions, as compared with 20% of patients receiving esas while on chemotherapy. Blood transfusions have a fairly high-safety profile, but the risk of infectious components continues to be a concern. The risk is always evolving as new infectious agents are found within human donor products. The risk of blood transfusions must constantly be weighed against their benefit.

### 2.2 Improvement in QOL

Other than minimizing the need for blood transfusions, one of the main reasons that physicians order esas for their patients on chemotherapy is to achieve an improvement in qol. As duration of survival increases, qol is increasingly being perceived as an important goal in the management of cancer. Symptoms of anemia include dyspnea, weakness, tachycardia, impaired cognition, depression, and fatigue. It is understandable how all of those symptoms may lead to diminished qol and how avoidance of anemia and subsequent symptoms may lead to improved qol.

When epoetin alfa and darbepoetin alfa were approved in Canada and the United States, they were approved for their statistically significant reduction in the requirement for transfusions. They did not meet criteria set out by the U.S. fda for approval for the indication of qol improvement. Those fda criteria are extensive, and they include 17

- use of validated instruments;
- double-blinding of personnel administering the qol questionnaires;
- prospective identification of key outcomes, critical time points, and minimum differences in scores to be considered clinically significant; and
- a detailed plan for preventing missing data, investigating the pattern of missing data, and addressing missing data in the analysis.

Although not meeting the foregoing criteria, multiple trials have reported a positive correlation between esa use and improvement in qol. The strongest evidence comes from a randomized controlled trial by Littlewood *et al.* 11. That trial enrolled 375 patients with a mean baseline hemoglobin of 10 g/dL. Of the 375 patients, 335 were evaluable for qol outcomes, which were evaluated using the Functional Assessment of Cancer Therapy–Anemia (fact-an) instrument and the Linear Analogue Self-Assessment (lasa) scales. Positive, statistically significant differences between the esa-treated and untreated groups were found. Results from the Medical Outcomes Study Short Form–36 (sf–36) showed a trend in the same direction, but did not reach significance (Table III). Criticism of the trial focused on missing descriptions of the key methodologic features for administering the qol instruments. As well, the study did not prospectively define the minimum changes in qol score that would be considered clinically significant.

Although not meeting fda requirements, three large community-based nonrandomized open-label studies performed with epoetin alfa can still provide insights. Those studies enrolled a total of 7724 patients, of whom 7283 were assessable. Epoetin alfa was administered to anemic patients with non-myeloid malignancies undergoing standard chemotherapy. Glaspy *et al.* 5 and Demetri *et al.* 6 used 3-times-weekly dosing, and the third study by Gabrilove *et al.* 7 used once-weekly dosing. Measures of qol included the fact-General, the fact-an subscale, and the lasa. The validated cancer-specific lasa instrument, which is used to assess therapy-related qol, measures the qol parameters of Energy Level, Ability to Do Daily Activities, and Overall QOL. In each of the three studies, the scores for the latter three items were significantly (*p* < 0.001) improved from baseline (Table IV). In the study by Glaspy and colleagues 5, increases in hemoglobin level were correlated with the improvements for each of the qol parameters independent of tumour response, and in the study by Demetri and colleagues 6, similar correlations were evident in patients having a complete response, a partial response, or stable disease.

Finally, the most recent Cochrane analysis included a systematic review of 9353 cancer patients enrolled into randomized controlled trials of epoetin alfa, epoetin beta, or darbepoetin alfa, updating the evidence to include trials from 1985 to 2005 18. The authors concluded that the results show an overall positive effect of epoetin on qol that seems unlikely to be attributable to chance.

### 2.3 Improvement in Cognition

The central nervous system has been demonstrated to contain epoetin receptors and to produce epoetin. Epoetin has been hypothesized to possibly protect neurons from injury such as ischemia, trauma, epilepsy, Parkinson disease, and disturbances of cognitive function 19. The ability of esas to improve cognition was demonstrated by O’Shaughnessy *et al.* 20 in women with breast cancer undergoing adjuvant chemotherapy. That study was followed by a randomized double-blind placebo-controlled trial to prospectively evaluate the effects of esas on several factors (cognitive function, mood, asthenia, and qol ) 21 Patients were randomized to receive 40,000 IU of subcutaneous epoetin alfa or placebo every week during 4 cycles of chemotherapy over 3 months. Interim results indicated that the use of esa improved hemoglobin and asthenia, reduced qol decline, improved mood, and may have ameliorated cognitive function. Because of the small sample size, the results for cognitive function were not statistically significant; they remain hypothesis-generating only.

## 3. RISKS

### 3.1 Increased Risk of Thrombosis

A significant side effect of drugs classified as esas is an increased risk of thromboembolic events. The recently published Cochrane meta-analysis included 9353 cancer patients enrolled from 1985 to 2005 into 57 randomized placebo-controlled trials using epoetin alfa, epoetin beta, or darbepoetin alfa 18. Treatment with an esa increased the risk of thromboembolic events [relative risk: 1.67; 95% confidence interval (ci): 1.35 to 2.06]. This risk increased proportionately as target hemoglobin rose. The causation of thromboembolic events in patients receiving erythropoietin growth factors is complex because of the increased baseline risk of thrombosis associated with chemotherapy and with cancer in general. Accepted guidelines must be adhered to, and careful follow-up of patients is mandatory.

### 3.2 Survival Analysis

With regard to survival, two early studies questioned a survival benefit for esas given to cancer patients. More recently, two additional trials demonstrated a negative effect on overall survival. These four negative trials that resulted in shorter survival deserve discussion.

The Breast Cancer Erythropoietin Survival Trial (best) 22 was a randomized trial in metastatic breast cancer. Patients who were not anemic received epoetin alfa or placebo, aiming to achieve a hemoglobin level of 12–14 g/dL. Decreased survival at 1 year [hazard ratio (hr): 1.35] was seen in the group treated with epoetin alfa, with most of the deaths occurring in the first 4 months, possibly secondary to thrombotic cardiac vascular events. The aim for higher-than-standard hemoglobin levels may explain this result and is now discouraged.

The enhance trial 23 was a study in patients with head-and-neck cancer randomized to either placebo or epoetin beta to achieve hemoglobin levels of 14.5–15 g/dL. Patients were not anemic, and they received radiation, but not chemotherapy. Again, a decrease in survival (hr: 1.39) was seen in the esa-treated group, who, like the best trial patients, were being treated to reach above-standard hemoglobin levels.

The epo-can 20 trial 24 was a randomized trial in non-small-cell lung cancer patients who were anemic. Patients studied were not on active treatment with either high-dose thoracic radiation or platinum-based chemotherapy. Because an increase in the risk of thrombosis was being recognized as feature of esas, an unplanned safety analysis was performed after 70 patients had been randomized. The median survival favoured the patients on the placebo arm (hr: 1.84), and the study closed. Given the early high mortality and the extremely small sample size, drawing any conclusions was hard. The application of the trial was also questioned, because esa use in cancer patients not on active treatment is not common practice.

Finally, Amgen 20010103 was a randomized trial of darbepoetin alfa in cancer patients who were anemic and, as in epo-can 20, not on active cancer therapy. Results were reported in letter format only in *The Cancer Letter* 25. The primary endpoint, reduction in transfusion, was not met, and an increase in mortality (hr: 1.30) was seen in the esa arm. The dosing regimen was not consistent with the approved darbepoetin alfa product monograph. Follow-up was only 4.3 months, and further follow-up and analysis will be ongoing. As was seen in epo-can 20, the use of esas in patients not on active treatment is not common practice and is now discouraged.

In summary, of the four negative survival trials, the first two treated patients so as to reach higher-than-standard hemoglobin levels, and the second two were conducted in cancer patients not on active treatment. All four were studying esas outside of the standard guidelines and indications.

Multiple randomized trials have been published showing no survival disadvantage with the use of esas. Most recently, in large randomized trials of lung cancer patients receiving chemotherapy, early versus late intervention with epoetin alfa 26 and a placebo-controlled comparison of darbepoetin 13 revealed no survival decrement. A randomized double-blind placebo-controlled trial of epoetin alfa in treatment of patients with small-cell lung cancer was published in the *Journal of Clinical Oncology* in 2005 27. That study also closed prematurely, not because of safety concerns, but because of low accrual. Baseline hemoglobin values were 12.8 g/dL and 13 g/dL in the two groups at the time of initiation of treatment. The primary endpoint—overall tumour response—was not significantly different at 72% for the epoetin alfa group and 67% for placebo. Hemoglobin was stable in the epoetin alfa group and decreased in the placebo group. Even though treatment with epoetin alfa was outside the current guidelines, survival in both groups was similar.

In terms of survival, the first Cochrane analysis reported inconclusive evidence that erythropoietin may improve overall survival (hr: 0.81; 95% ci: 0.67 to 0.99). Trials through December 2001 were included. The more recent analysis that incorporated trials up to 2005 included the best, enhance, and epo-can 20 studies. This latter analysis resulted in a hazard ratio of 1.08 (95% ci: 0.99 to 1.18). The authors concluded that there was uncertainty regarding “whether and how epoetin or darbepoetin effects overall survival” 18.

### 3.3 Tumour Progression

Finally, tumour progression secondary to activation of erythropoietin receptors has been questioned 28. Both best and enhance showed decreased tumour control, but as discussed earlier, both used unapproved dosing regimens. A third study deserves mention. The dahanca trial 29 followed the enhance study and was designed to avoid the numerous protocol violations in enhance and subsequent difficulty in interpreting the results. As a prospective trial in head-and-neck squamous cell carcinoma in patients who were undergoing treatment with definitive radiotherapy, dahanca planned to randomize 600 patients to darbepoetin alfa or placebo. Its primary objective was the 5-year locoregional control rate. An interim analysis in 484 patients demonstrated a 10% increase in the locoregional failure rate among patients treated with darbepoetin alfa (*p* = 0.01). Overall survival was not significantly different, but did trend toward shorter survival in the esa arm (*p* = 0.08). This study had many limitations. The dosing regimen and dose adjustment rules for darbepoetin alfa were not approved. The target hemoglobin was 14–15.5 g/dL. As well, the design of the study was not adequate to assess tumour proliferation, because no uniform imaging assessment was conducted at baseline or at recurrence, nor was proliferation confirmed by biopsy. To further complicate the interpretation of study results, patients were treated with a hypoxic radiosensitizer, nimorazole, which is not routine practice. Moreover, nimorazole has not been studied for safety when given in combination s with esa 30. The final study analysis will be reported to the fda in late 2008.

The relationship between the presence of erythropoietin receptors and tumour proliferation attributable to exogenous erythropoietin with the use of esas has not been established. Outside of simple presence, the function of erythropoietin receptors is not well understood. *In vitro* studies vary in their conclusions. If erythropoietin has a direct effect on tumour cell growth, then cancer cells must express functional erythropoietin receptors or protein on their surface. The commercial antibody used to detect such receptors is Santa-Cruz C-20. This antibody lacks specificity and cannot distinguish cell-surface expression from intracellular expression 31. The results of further studies must be awaited.

In summary, the risk of thrombosis and embolism is definitely increased with the use of esas. Careful monitoring of hemoglobin levels in patients is mandatory. The negative survival studies are difficult to interpret because of target hemoglobin levels higher than those recommended, poor study design, and enrolment of patients not on active care. The trials showing tumour progression suffer from similar limitations.

## 4. SUMMARY

Cancer-related anemia impairs patient functioning through a variety of direct and indirect mechanisms. A large body of evidence demonstrates that treatment with esas of anemia in cancer patients on active chemotherapy significantly increases hemoglobin levels, reduces the requirement for transfusions, and improves qol. In addition, gains in cognitive function are hypothesized.

Risks are also associated with esas: Safety issues include increased thromboembolic risk. As well, four studies show decreased survival and three studies suggest tumour proliferation with the use of esas. All of these findings are limited, because all of the studies used unapproved dosing regimens or aimed for hemoglobin levels higher than generally recommended. Attention to the target hemoglobin of 12 g/dL must be emphasized. Adherence to guidelines concerning esas in the management of cancer patients on active treatment should be mandatory.

In May 2004, the fda’s Oncologic Drugs Advisory Committee (odac) revised product labelling to include warnings against maintaining hemoglobin levels above 12 g/dL. Given the decreased survival seen in the Amgen 20010103 study discussed earlier, the odac met again in May 2007. “Black box” warnings were initiated in both Canada and the United States. The panel voted to have Amgen and Johnson & Johnson further strengthen the warning labels of their esas and carry out additional safety studies on the drugs. A final report from the fda is still pending.

## Figures and Tables

**TABLE I:** Proportion of patients on epoetin alfa (epo) transfused during chemotherapy

Chemotherapy regimen	Patients transfused (%)			
	On study		During months 2 and 3	
	epo	Placebo	epo	Placebo
Without cisplatin	44	44	21	33
With cisplatin	50	63	23 *p* < 0.05	56
Combined	47	53	22 *p* < 0.05	43

**TABLE II:** Proportion of patients on darbepoetin alfa transfused during chemotherapy

Period	Patients transfused			
	Darbepoetin alfa		Placebo	
	(*n*)	*(%)*	(*n*)	*(%)*
Week 5 to end of treatment	31	27	77	52
	(*N =* 148)		(*N =* 149)	

**TABLE III:** Quality of life (qol) outcomes in Littlewood *et al.* 11

	Evaluable for		Overall qol		Energy level		Daily activities			Other qol	
Treatment arm	Transfusion (*n*)	qol (*n*)	% Change	*p* Value	% Change	*p* Value	% Change	*p* Value	Other qol measure	% Change	*p* Value
Control	115	108	na		−5.8		−6.0				
Epoetin	244	227	na	<0.01	7.8	<0.001	7.3	<0.01			
Control	115	90							fact-an: anemia	−9.4	
Epoetin	244	200							fact-an: anemia	14.4	<0.01
Control	115	90							fact-an: fatigue	−4.2	
Epoetin	244	200							fact-an: fatigue	5.7	<0.01
Control	115	na							sf-36	na	
Epoetin	244	na							sf-36	na	ns

na = not available; fact-an = Functional Assessment of Cancer Therapy–Anemia; sf-36 = Medical Outcomes Study Short Form–36.

**TABLE IV:** Quality of life (qol) outcomes a in community-based studies

qol parameter	Mean change in lasa score from baseline [mm (%)]		
	Glaspy et al., 1997 5 (*n* = 1498)	Demetri et al., 1998 6 (*n* = 1749)	Gabrilove et al., 2001 7 (*n* = 2258)
Energy level	15.0 (38)	11.5 (29)	11.9 (30)
Ability in daily activities	13.9 (32)	11.2 (28)	10.8 (27)
Overall qol	11.0 (24)	9.8 (21)	9.3 (20)

a All results in this table: *p* < 0.01 versus baseline.
